# Galantamine as a Treatment Option for Nicotine Addiction

**DOI:** 10.1155/2021/9975811

**Published:** 2021-07-17

**Authors:** Qutub Jamali

**Affiliations:** Lancashire & South Cumbria NHS Foundation Trust, UK

## Abstract

The pharmacological therapy for smoking cessation recommended by National Institute for Health and care Excellence (NICE) guidelines is nicotine replacement therapy such as gum, inhalator, lozenge, nasal spray, oral spray, sublingual tablet, and transdermal patch. Medications such as bupropion and varenicline are also used. Varenicline is the only established drug used to alleviate symptoms of craving as it acts as a partial nicotine agonist. Galantamine has a similar mechanism of action where it is an acetylcholinesterase inhibitor and nicotinic receptor agonist. However, varenicline is the only recommended drug. There are not many studies to illustrate the effectiveness of galantamine for smoking cessation. This article explores the possibility of potential use of galantamine in alleviating the symptoms of nicotine withdrawal.

## 1. Introduction

Smoking is a major risk factor for several medical conditions. According to a study by West et al. [1], the prevalence of smoking has risen in England by 5.5% in 2020 [1]. According to the study, in 2019, the estimated prevalence of smoking among adults in England was 29.1%. By July 2020, the prevalence was 34.6%. However, the success rate of quitting smoking had increased in 2020 (22.9%) compared to 2019 (14.2%) by 8.7% [1]. Public Health England in 2020 reported that, while a reduction in smoking rates has been noticed among adults with long-term mental health illness, a drop of 8.5% from 35.3% in 2013-14 to 26.8% in 2018-19, the prevalence remains significantly higher, despite equal levels of motivation to quit [2]. In 2019, the prevalence rate of smoking among adults with long-term mental health disorders is 26.8% compared to 14.5% among adults aged 18 and above [2]. The pharmacological therapy for smoking cessation recommended by National Institute for Health and Care Excellence (NICE) Guidelines (2018) is nicotine replacement therapy such as gum, inhalator, lozenges, nasal spray, oral spray, sublingual tablets, and transdermal patch [3]. Moreover, medications such as bupropion and varenicline are also used [3]. According to NICE guidelines (2018), the most common withdrawal symptom following smoking cessation is nicotine craving where 70% of people are affected [4]. Currently, varenicline is the only established drug used to alleviate symptoms of craving [3]. This article will focus on another agent which could potentially be used to reduce nicotine cravings.

## 2. Main Text

When a person inhales smoke from a cigarette, nicotine gets into the lungs and is absorbed into the pulmonary venous circulation and enters the arterial circulation [5]. Nicotine then moves rapidly to the brain and binds to nicotinic cholinergic receptors (nACHRs), particularly the *α*4*β*2 receptor [5]. Stimulation of nACHRs releases neurotransmitters in the brain such as acetylcholine and dopamine. When dopamine is released in a large amounts, it is associated with intense pleasure and a sense of relief leading to nicotine addiction [5]. Varenicline is a selective partial agonist which acts on nACH receptors including *α*4*β*2 [6]. It is useful to understand the difference between full and partial nicotine agonists. A full nicotine agonist will cause substantial dopamine release. However, a partial nicotine agonist such as varenicline will release dopamine to a lesser extent than a full agonist [6]. Moreover, the partial antagonist action will block the effects of any nicotine entering the system [5]. It activates the receptors at lower concentrations, which is helpful in smoking cessation in two ways. Firstly, the partial agonist action maintains moderate levels of dopamine to counteract the withdrawal symptoms. Secondly, partial antagonist activity reduces smoking satisfaction [6]. Galantamine is an acetylcholinesterase inhibitor and nicotinic receptor agonist [7]. It is widely recommended and used as a cognitive enhancer as it inhibits acetylcholinesterase which leads to prolonged exposure of acetylcholine in the brain [7]. This may be useful in reducing the cognitive deficits associated with nicotine withdrawal [7]. Moreover, since it is also a nicotine receptor agonist, it acts as an allosteric (alteration of the activity) positive modulator of nACH receptors [7]. The binding sites of full nicotine agonists are between the *α* and *β* subunits. However, due to the allosteric function, galantamine binds only to *α* subunit which includes *α*4*β*2 [7]. It has around 90% bioavailability and has a relatively large volume of distribution and low protein binding [8]. Metabolism is mainly through the cytochrome P450 system, specifically the CYP2D6 and CYP3A4 isoenzymes [8]. Polycyclic aromatic hydrocarbons (PAHs) of tobacco smoke interact with cytochrome 450 (CYP) enzymes such as CYP1A1, CYP1A2, possibly CYP2E1, and Uridine diphosphate-glucuronosyl transferase (UGTs) enzymes [9]. The medications metabolized by CYP1A1, CYP1A2, CYP2E1, and UGT enzymes might be affected by tobacco smoking and require higher doses to be prescribed due to decreased plasma concentrations through increased induction by PAHs of tobacco smoke [9]. Since galantamine is mainly metabolized through CYP2D6 and CYP3A4 [8], it may not interact with PAHs.

Based on the description above, varenicline and galantamine appear to have similar mechanism of action in alleviating the symptoms of nicotine withdrawal. However, varenicline is the recommended drug of choice over galantamine for smoking cessation. A study conducted by Ashare et al. in 2016 indicated that galantamine treatment reduces smoking satisfaction and rewards with minimal side effects [10]. Maclean et al. [11] reported that galantamine reduces smoking behaviour if started prior to a quit attempt [11]. If we compare the side effect profile of varenicline and galantamine, both have cardiovascular side effects and are recommended for use with caution. Varenicline is sometimes underutilised due to its cardiovascular and psychiatric side effects [3]. However, there is no caution in place for the use of galantamine in people having mental health problems [12]. A randomized trial by Sangaleti et al. [13] found that galantamine treatment reduces oxidative stress by increasing antioxidant enzyme activities which includes superoxide dismutase (SOD), catalase (CAT), and glutathione peroxidase [13]. Moreover, galantamine significantly alleviates insulin resistance reducing the risk of metabolic syndrome and cardiovascular diseases [13].

Several randomized controlled trials have been conducted to determine the effectiveness of varenicline for smoking cessation compared with placebo. However, there are not many studies to illustrate the effectiveness of galantamine for smoking cessation. A randomized, placebo-controlled trial conducted by Diehl et al. [14] suggested that in 114 randomized smokers who received galantamine (*n* = 56) or placebo (*n* = 58) for 12 weeks, there was 20% reduction in cumulative number of cigarettes smoked in the galantamine group compared to placebo [14]. Moreover, the average number of smoked cigarettes per smoking day as well as the cotinine values reduced by 10% in those on galantamine compared to placebo [14]. However, the aim should be to abstain from smoking rather than reducing the number of cigarettes and tobacco intake [3]. It would be interesting to do a randomized double-blind controlled trial comparing varenicline with galantamine to determine the effectiveness as both have similar mechanisms of action. The duration of treatment of varenicline is 12 weeks, which begins two weeks before the quit date. It can be prescribed for another 12 weeks to prevent relapse. However, limited evidence suggests that varenicline can contribute to relapse prevention [15]. As far as galantamine is concerned, it is generally prescribed lifelong as a cognitive enhancer in dementia. There is a potential for galantamine to be prescribed as a relapse prevention treatment in smoking cessation.

## 3. Conclusion

Recommended pharmacological therapies for smoking cessation include nicotine replacement therapy, bupropion, and varenicline. According to NICE guidelines (2013), it is important to “identify people who smoke at the first opportunity advising them to stop, providing pharmacotherapy to support abstinence, offering intensive behavioural support and following up with them at the next opportunity” [16]. Galantamine could potentially be another useful addition to pharmacotherapy as a relapse prevention treatment in smoking cessation. Further clinical trials with a larger sample size are required to establish the effectiveness of galantamine as a treatment option for nicotine addiction.
